# Short-term outcomes of Early *versus* conventional adjuvant chemotherapy in stage III colon cancer: randomized clinical trial

**DOI:** 10.1093/bjsopen/zrad064

**Published:** 2023-07-13

**Authors:** Kyung Ha Lee, Soo Yeun Park, Seung Ho Song, Hye Jin Kim, Jong Gwang Kim, Byung Woog Kang, In Kyu Lee, Yoon Suk Lee, So Hyun Kim, Seong Kyu Baek, Sung Uk Bae, Gyung Mo Son, Ki Beom Bae, Gyu-Seog Choi, Jun Seok Park, Ji Yeon Kim

**Affiliations:** Department of Surgery, Chungnam National University Hospital, Chungnam National University College of Medicine, Daejeon, Korea; Colorectal Cancer Centre, Kyungpook National University Medical Centre, School of Medicine, Kyungpook National University, Daegu, Korea; Colorectal Cancer Centre, Kyungpook National University Medical Centre, School of Medicine, Kyungpook National University, Daegu, Korea; Colorectal Cancer Centre, Kyungpook National University Medical Centre, School of Medicine, Kyungpook National University, Daegu, Korea; Department of Oncology/Haematology, Kyungpook National University Medical Centre, Kyungpook National University School of Medicine, Daegu, Korea; Department of Oncology/Haematology, Kyungpook National University Medical Centre, Kyungpook National University School of Medicine, Daegu, Korea; Department of Surgery, Seoul St Mary’s Hospital, College of Medicine, The Catholic University of Korea, Seoul, Korea; Department of Surgery, Seoul St Mary’s Hospital, College of Medicine, The Catholic University of Korea, Seoul, Korea; Division of Colon and Rectum, Department of Surgery, College of Medicine, Yeungnam University, Daegu, Korea; Department of Surgery, School of Medicine, Dongsan Medical Centre, Keimyung University, Daegu, Korea; Department of Surgery, School of Medicine, Dongsan Medical Centre, Keimyung University, Daegu, Korea; Department of Surgery, Busan National University, Busan, Korea; Department of Surgery, Busan Paik Hospital, Inje University, Busan, Korea; Colorectal Cancer Centre, Kyungpook National University Medical Centre, School of Medicine, Kyungpook National University, Daegu, Korea; Colorectal Cancer Centre, Kyungpook National University Medical Centre, School of Medicine, Kyungpook National University, Daegu, Korea; Department of Surgery, Chungnam National University Hospital, Chungnam National University College of Medicine, Daejeon, Korea

## Abstract

**Background:**

Evidence is lacking regarding the earliest timing of initiating adjuvant chemotherapy to maximize its efficacy safely. A trial was designed and conducted to evaluate the safety and oncological efficacy of early adjuvant chemotherapy compared with conventional adjuvant chemotherapy. The short-term outcomes are reported here.

**Methods:**

A multicentre, randomized (1 : 1), open-label, phase III trial was conducted comparing early adjuvant chemotherapy with conventional adjuvant chemotherapy in patients with stage III colon cancer. Patients who underwent radical surgery who had stage III colon cancer confirmed by histopathological assessment were screened and randomized into the early adjuvant chemotherapy arm or the conventional adjuvant chemotherapy arm. The primary endpoint was 3-year disease-free survival. The adjuvant chemotherapy with FOLFOX was delivered between postoperative day 10 and 14 in the early adjuvant chemotherapy arm, and between postoperative day 24 and 28 in the conventional adjuvant chemotherapy arm. Toxicity and quality of life were evaluated.

**Results:**

Between 9 September 2011 and 6 March 2020, 443 patients consented to randomization at eight sites. The intention-to-treat population included 423 patients (209 in the early adjuvant chemotherapy arm and 214 in the conventional adjuvant chemotherapy arm), and the safety population included 380 patients (192 in the early adjuvant chemotherapy arm and 188 in the conventional adjuvant chemotherapy arm). There was no statistically significant difference in overall toxicity (28.1 per cent in the early adjuvant chemotherapy arm and 28.2 per cent in the conventional adjuvant chemotherapy arm, *P* = 0.244), surgical complications, and quality of life between the two arms.

**Conclusion:**

Adjuvant chemotherapy can be safely initiated 2 weeks after surgery with toxicity and quality of life comparable to conventional adjuvant chemotherapy for stage III colon cancer.

## Teaser

The aim of this study was to evaluate the safety of early initiation of adjuvant chemotherapy within 2 weeks after surgery in patients with stage III colon cancer. Findings revealed that there was no statistically significant difference in overall toxicity, surgical complications, and quality of life between the early and conventional adjuvant chemotherapy arms.

## Introduction

Adjuvant chemotherapy (AC) after radical surgery is the current standard treatment for high-risk stage II and III colon cancer with a survival benefit due to eradication of micrometastases^1–3^. Initiating AC beyond 8 weeks after surgery is associated with worse overall survival (OS) and disease-free survival (DFS)^4–13^. It is recommended that AC is initiated within 8 weeks after surgery; however, the earliest time that it can safely be commenced is not yet defined.

Many preclinical studies suggest that surgery due to manipulation of the tumour may affect tumour kinetics, facilitate circulation of tumour cells, and increase metastatic potential^14^. A postoperative increase in angiogenesis and oncogenic growth factors in immunocompromised status can potentiate the movement and growth of residual tumour cells^15–17^. Therefore, theoretically, AC should be initiated as soon as possible after surgery to maximize its efficacy.

Although the negative impact of delaying AC is evident, initiating AC immediately after surgery for colon cancer has been avoided. This is due to concerns that cytotoxic agents can compromise tissue healing of wounds and the anastomosis. Patients need enough time to recover to be able to tolerate cytotoxic therapy safely. However, the postoperative recovery interval has been reduced due to minimally invasive surgery and Enhanced Recovery After Surgery (ERAS) protocols. Therefore, more patients should be physiologically able to tolerate AC earlier than in the past.

Most previous studies have focused on how long you can delay AC rather than how early is safe. Therefore, a prospective randomized trial was designed and conducted to evaluate the safety and oncological efficacy of early AC (EAC) compared with conventional AC (CAC). In this study, the short-term outcomes of this trial are reported.

## Methods

### Study design and participants

A multicentre, randomized (1 : 1), open-label, phase III trial was conducted comparing EAC with CAC in patients with stage III colon cancer. Patients were recruited from eight centres in South Korea. All centres were tertiary medical institutes with sub-specialist colorectal surgeons. Patients with stage III colon cancer were screened after surgery to determine whether they met the inclusion criteria: age greater than or equal to 18 years with Eastern Cooperative Oncology Group (ECOG) performance status 0–2; histologically confirmed adenocarcinoma of the colon (tumours greater than 12 cm from the anal verge or above the peritoneal reflection); undergone standard, minimally invasive, curative R0 resection with D3 lymphadenectomy; discharged within 10 days after surgery; stage III based on the Seventh Edition of the AJCC Cancer Staging Manual; fully recovered hepatic, renal, and haematological function, as assessed by serum chemistry with calculated creatinine clearance, liver function test, and full blood cell count; and able to understand and willing to consent. The main exclusion criteria were: rectal cancer; metastatic or radically unresectable disease; stage I or II colon cancer based on the Seventh Edition of the AJCC Cancer Staging Manual; any contraindication for chemotherapy, including age greater than 85 years or life expectancy under 5 years due to non-cancer-related disease; hypersensitivity to treatment component(s); unable to be discharged 10 days after surgery due to any postoperative complications; emergency operation for tumour obstruction or perforation; history or presence of synchronous malignancy; previous chemotherapy; and being pregnant or breastfeeding.

The study was conducted as per the Declaration of Helsinki and the Good Clinical Practice guidelines. The study protocol was approved by the Kyungpook National University Hospital Ethics Committee (version 1.3) on 21 January 2012, and its equivalent in other participating institutions. All participants provided written informed consent before enrolment. Trial oversight was maintained by a combined trial steering committee and a data monitoring committee. This study was registered at ClinicalTrials.gov (NCT01460589).

### Randomization and procedure protocol

The anaesthetic evaluation and patient information regarding the operative procedure were performed according to the local practices of each investigation centre. Radical surgery was performed as per the oncological quality criteria for resection. Preoperative and postoperative data were reported on specific forms. If the histological assessment confirmed stage III disease and the patient met the inclusion criteria, eligible patients were randomly assigned (1 : 1) to the EAC arm or the CAC arm. The randomization sequence was concealed from the investigators, and randomization was performed using a web-based software platform (Velos, Fermont, CA, USA) and centrally coordinated by the Clinical Research Coordination Centre of the Kyungpook National University Cancer Centre (Daegu, Korea).

The assigned AC was delivered between postoperative day (POD) 10 and 14 in the EAC arm, and between POD 24 and 28 in the CAC arm. The AC regimen was FOLFOX, which comprised oxaliplatin 85 mg/m^2^, leucovorin 200 mg/m^2^, and bolus fluorouracil 400 mg/m^2^ on day 1, and infusion of fluorouracil 2400 mg/m^2^ over 46 h, every 2 weeks, for a total of 12 cycles. If clinically indicated based on the investigator’s discretion, the doses of chemotherapeutic drugs were reduced by 75 per cent at the start.

Toxicity was evaluated based on Common Terminology Criteria for Adverse Events (CTCAE) 4.0, and dose modifications were based on the most severe adverse events and the investigator’s discretion. Adverse events were monitored during and after the study treatment, and a complete laboratory examination was performed on day 1 of each treatment cycle. For treatment-related adverse events of grade 1, treatment was continued at the total dose. For grade 2, treatment was withheld and restarted after recovery to grade 1. The dose was reduced for grade 3 adverse events and grade 4 stomatitis with a delay in the treatment schedule, if necessary. Treatment was discontinued in the event of any documented disease recurrence, grade 4 or 5 adverse events, or a patient’s refusal.

After completing the protocol treatment, patients were followed up according to a predefined surveillance schedule until recurrence, development of another malignancy, or death. Carcinoembryonic antigen level assessment with a general blood test and abdominopelvic CT scans were performed every 3 to 6 months for the first 2 years and every 6 months thereafter. Chest CT scans were performed every 8 to 12 months. A colonoscopy was routinely performed 1 year and 5 years after surgery.

### Endpoints

The primary endpoint was 3-year DFS, defined as the time from the date of the operation to the earliest date of recurrence.

The secondary endpoint was OS, defined as the time from the date of the operation to the date of death due to all causes or the date of the last follow-up, chemotherapy-related adverse events according to CTCAE 4.0, surgical complications during chemotherapy according to the Clavien–Dindo classification, and quality of Life (QoL) assessment according to the European Organization for Research and Treatment of Cancer Quality of Life Questionnaire (EORTC QLQ) C30. The patients completed baseline questionnaires after providing consent and before initiating AC. When patients visited the outpatient clinic, follow-up questionnaires were administered to patients 1, 3, 6, and 12 months after surgery.

The review of case report forms was conducted annually for quality control by the Kyungpook National University Cancer Centre (Daegu, Korea).

### Statistics

The expected 3-year DFS with CAC was 72 per cent based on a literature review^9^. A 10 per cent gain in 3-year DFS was predicted in the EAC arm compared with the CAC arm (82 *versus* 72 per cent respectively). For a statistical power of 80 per cent for the superiority hypothesis at a one-sided significance level of 0.05, the sample size was calculated as 220 patients in each arm, assuming that 14 per cent of patients were lost to follow-up. PASS 11™ software (NCSS, Kaysville, UT, USA) was used to compute the sample size.

All randomized patients were defined as the intention-to-treat (ITT) population. Patients with protocol violations, which meant the date of initiating chemotherapy did not meet the protocol, were excluded from the per-protocol population. Safety analyses were performed on the safety population, which meant all patients who received at least one cycle of chemotherapy after randomization. QoL analyses were performed on patients in the safety population who answered QoL questionnaires at baseline and follow-up.

The χ^2^ test or Fisher’s exact test was used for comparing categorical variables, and Student’s *t* test or the Mann–Whitney *U* test was used to compare continuous variables, depending on the distribution. The outcomes of EORTC QLQ C30 were described using means and standard errors of means. Questionnaire responses were collected five times from baseline to 12 months after surgery. Patients who completed at least one questionnaire response after the baseline time point were included in the analysis irrespective of the interval. A mixed model for repeated measures (MMRM) with the assumption of an unconstructed covariance structure for repeated measurements was applied for the analysis. The least-square mean estimates were calculated for post-hoc analysis to compare the two arms at each interval.

Statistical analyses were performed using SPSS^®^ (IBM, Armonk, NY, USA; version 26). All *P* values were reported as two-sided and results with *P* < 0.050 were considered statistically significant.

## Results

### Patient and disease characteristics

**Fig. 1:**
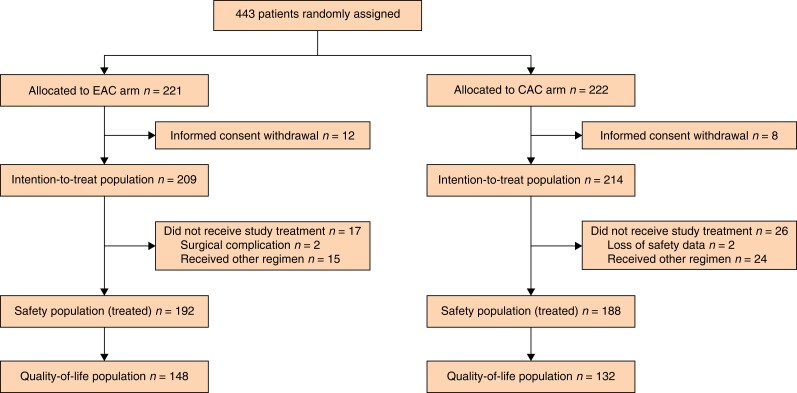
Flow diagram of study population EAC, early adjuvant chemotherapy; CAC, conventional adjuvant chemotherapy.

Between 9 September 2011 and 6 March 6 2020, 443 patients consented to randomization at eight sites; 221 and 222 patients were allocated to the EAC arm and the CAC arm respectively (*Fig. 1*). After randomization, 20 patients (12 in the EAC arm and 8 in the CAC arm) withdrew consent. The ITT population included 423 patients (209 in the EAC arm and 214 in the CAC arm).

A total of 43 patients (17 in the EAC arm and 26 in the CAC arm) did not receive treatment as per the study protocol. In the EAC arm, two patients could not initiate AC due to the late surgical complications, and 39 patients (15 in the EAC arm and 24 in the CAC arm) received other regimens for AC, including intravenous fluorouracil/leucovorin (FL), oral capecitabine, and oxaliplatin with oral capecitabine. The safety population included 380 patients (192 in the EAC arm and 188 in the CAC arm) because safety data were missing for 2 patients in the CAC arm. The QoL population included 280 patients (148 in the EAC arm and 132 in the CAC arm) because QoL data were missing for 100 patients (44 in the EAC arm and 56 in the CAC arm) among the safety population.

The baseline and pathological characteristics of the ITT population, the safety population, and the QoL population are shown in *Table 1*. All patient and tumour characteristics were evenly distributed in the two arms in all populations.

**Table 1:** Baseline characteristics

	Intention-to-treat population			Safety population			QoL population		
	EAC (*n* = 217)	CAC (*n* = 221)	*P*	EAC (*n* = 192)	CAC (*n* = 188)	*P*	EAC (*n* = 148)	CAC (*n* = 132)	*P*
**Age (years), median (range)**	61.0 (32–83)	61.0 (28–83)	0.285	60.0 (32–80)	60.0 (28–83)	0.764	60.5 (32–80)	61.5 (28–83)	0.213
**Sex**			0.977			0.729			0.089
Male	125 (57.6)	127 (57.5)		110 (57.3)	111 (59.0)		77 (52.0)	82 (62.1)	
Female	92 (42.4)	94 (42.5)		82 (42.7)	77 (41.0)		71 (48.0)	50 (37.9)	
**ECOG performance status**			0.061			0.092			0.367
0	78 (35.9)	61 (27.6)		74 (38.5)	57 (30.3)		73 (49.3)	58 (43.9)	
1	139 (64.1)	160 (72.4)		118 (61.5)	131 (69.7)		75 (50.7)	74 (56.1)	
**Primary tumour location**			0.313			0.320			0.335
Ascending colon	44 (20.3)	43 (19.5)		38 (19.8)	31 (16.5)		25 (16.9)	22 (16.7)	
Hepatic flexure	6 (2.8)	14 (6.3)		6 (3.1)	13 (6.9)		5 (3.4)	6 (4.5)	
Transverse colon	9 (4.1)	14 (6.3)		9 (4.7)	13 (6.9)		6 (4.1)	9 (6.8)	
Splenic flexure	6 (2.8)	4 (1.7)		6 (3.1)	3 (1.6)		6 (4.1)	2 (1.5)	
Descending colon	18 (8.3)	10 (4.5)		16 (8.3)	9 (4.8)		13 (8.8)	4 (3.0)	
Sigmoid colon	75 (34.6)	79 (35.7)		68 (35.4)	71 (18.7)		56 (50.9)	54 (40.9)	
Rectosigmoid colon	59 (27.2)	57 (25.8)		49 (25.5)	48 (25.5)		37 (25.0)	35 (26.5)	
**Histological type**			0.072			0.159			0.333
Adenocarcinoma	143 (60.8)	154 (69.7)		114 (59.4)	125 (66.5)		72 (48.6)	69 (52.3)	
Mucinous adenocarcinoma	84 (38.7)	63 (28.5)		77 (40.1)	59 (31.4)		75 (50.7)	59 (44.7)	
Other	0 (0.0)	2 (0.9)		0 (0.0)	2 (1.1)		0 (0.0)	2 (1.5)	
Not recorded	1 (0.5)	2 (0.9)		1 (0.5)	2 (1.1)		1 (0.7)	2 (1.5)	
**Stage**			0.800			0.698			0.220
II	13 (6.0)	12 (5.4)		12 (6.3)	10 (5.3)		4 (2.7)	1 (0.8)	
III	204 (94.0)	209 (94.6)		180 (93.8)	178 (94.7)		144 (52.4)	131 (99.2)	
**Pathological T category**			0.673			0.611			0.668
Tx	0 (0.0)	2 (0.9)		0 (0.0)	2 (1.1)		0 (0.0)	1 (0.8)	
T1	11 (5.1)	9 (4.1)		11 (5.7)	8 (4.3)		9 (6.1)	7 (5.3)	
T2	13 (6.0)	13 (5.9)		12 (6.3)	12 (6.4)		11 (7.4)	9 (6.8)	
T3	150 (69.1)	150 (67.9)		134 (69.8)	128 (68.1)		103 (69.6)	86 (65.2)	
T4	43 (19.8)	47 (21.3)		35 (18.2)	38 (20.2)		25 (16.9)	29 (22.0)	
**Pathological N category**			0.444			0.620			0.399
N0	13 (6.0)	12 (5.4)		12 (6.3)	10 (5.3)		4 (2.7)	1 (0.8)	
N1	141 (65.0)	156 (70.6)		129 (67.2)	135 (71.8)		101 (68.2)	96 (72.7)	
N2	63 (29.0)	53 (24.0)		51 (26.6)	43 (22.9)		43 (29.1)	35 (26.5)	

Values are *n* (%) unless otherwise indicated. QoL, quality of life; EAC, early adjuvant chemotherapy; CAC, conventional adjuvant chemotherapy; ECOG, Eastern Cooperative Oncology Group.

### Results of adjuvant chemotherapy and toxicity

The median times from operation to initiating AC were 13 (range 4–43) days in the EAC arm and 29 (range 17–53) days in the CAC arm (safety population) (*P* < 0.001). In the QoL population these were 13 (range 10–14) days in the EAC arm and 28 (range 24–28) days in the CAC arm (*P* < 0.001) (*Table 2*). In the safety population, 27 patients (14 per cent) in the EAC arm initiated AC after 14 days (postoperative general weakness in 5 patients, late visit in 12 patients, and delay of the appointment with an oncologist or vascular access in ten patients).

**Table 2:** Results of adjuvant chemotherapy

	Safety population			QoL population		
	EAC (*n* = 192)	CAC (*n* = 188)	*P*	EAC (*n* = 148)	CAC (*n* = 132)	*P*
Days from surgery to chemotherapy, median (range)	13 (4–43)	29 (17–53)	<0.001	13 (10–14)	28 (24–28)	<0.001
Chemotherapy cycle, median (range)	12 (1–12)	12 (1–12)	1.000	12 (1–12)	12 (5–12)	1.000
**Completeness of treatment**			0.870			0.559
Completed without dose reduction or delay	35 (18.2)	38 (20.2)		23 (15.5)	28 (21.2)	
Completed with dose reduction or delay	125 (65.1)	114 (60.6)		94 (63.5)	79 (59.8)	
Completed FL with discontinuation of oxaliplatin	5 (2.6)	9 (4.8)		5 (3.4)	6 (4.5)	
Discontinued	27 (14.1)	27 (14.1)		26 (17.6)	19 (14.4)	
Toxicity of chemotherapy, *n*	16	15		16	12	
Patient refusal, *n*	7	3		7	3	
Drug allergy, *n*	3	1		2	0	
Recurrence, *n*	0	5		0	2	
Vascular port malfunction, *n*	0	2		0	1	
Others, *n*	1	1		1	1	
**Surgical complications during chemotherapy**	2 (1.0)	0 (0.0)	0.161	2 (1.4)	0 (0.0)	0.180
Anastomotic leakage	1 (0.5)	0 (0.0)		1 (0.7)	0 (0.0)	
Anastomotic stricture	1 (0.5)	0 (0.0)		1 (0.7)	0 (0.0)	

Values are *n* (%) unless otherwise indicated. QoL, quality of life; EAC, early adjuvant chemotherapy; CAC, conventional adjuvant chemotherapy; FL, fluorouracil/leucovorin.

The median number of administered cycles and completeness of chemotherapy, including the rate of completion without dose reduction or delay, the rate of completion with dose reduction or delay, the rate of completion of FL with discontinuation of oxaliplatin, and the rate of discontinuation of chemotherapy, were similar between the two arms. The most common reason for discontinuation of chemotherapy was toxicity in both arms, and five patients in the CAC arm had to discontinue the study protocol treatment and change to another chemotherapeutic regimen due to recurrence, compared with no recurrence during AC in the EAC arm.

Although there were two cases of postoperative surgical complications in the EAC arm (one case of anastomotic leakage and one case of anastomotic stricture) compared with none in the CAC arm, there was no statistically significant difference (1.0 per cent in the EAC arm *versus* 0.0 per cent in the CAC arm, *P* = 0.870).

The relative dose intensities of oxaliplatin in the two arms were similar and decreased to 80 per cent at the seventh cycle of AC in both arms (*Table 3*).

**Table 3:** Relative oxaliplatin dose intensity during each cycle (safety population)

Cycle	EAC		CAC		*P*
	Median dose (mg)	Relative dose intensity (%)*, median (range)	Median dose (mg)	Relative dose intensity (%)*, median (range)	
1	192	100 (70–100)	188	100 (70–100)	1.000
2	191	100 (70–100)	186	100 (70–100)	1.000
3	186	100 (70–100)	184	100 (60–100)	1.000
4	183	100 (60–100)	183	100 (60–100)	1.000
5	183	100 (60–100)	183	100 (60–100)	1.000
6	181	100 (50–100)	180	100 (60–100)	1.000
7	180	80 (50–100)	175	80 (60–100)	0.938
8	179	80 (50–100)	171	80 (60–100)	0.888
9	173	80 (50–100)	171	80 (60–100)	0.890
10	170	80 (60–100)	163	80 (60–100)	0.766
11	164	80 (50–100)	156	80 (60–100)	0.943
12	160	80 (50–100)	152	80 (60–100)	0.952

*The ratio of the median of the actual dose to the initial dose that is recommended by guidelines. EAC, early adjuvant chemotherapy; CAC, conventional adjuvant chemotherapy,

Details of toxicity are presented in *Table S1*. The percentage of patients who experienced grade 3 or higher toxicity was similar (28.1 per cent in the EAC arm and 28.2 per cent in the CAC arm, *P* = 0.244). There was no difference in the overall or individual toxicity between the two arms, except for alopecia. The rate of grade 1–2 alopecia was higher in the EAC arm (11 per cent *versus* 5 per cent in the CAC arm, *P* = 0.045), but there was no grade 3 alopecia in either arm.

### Quality of life

**Fig. 2:**
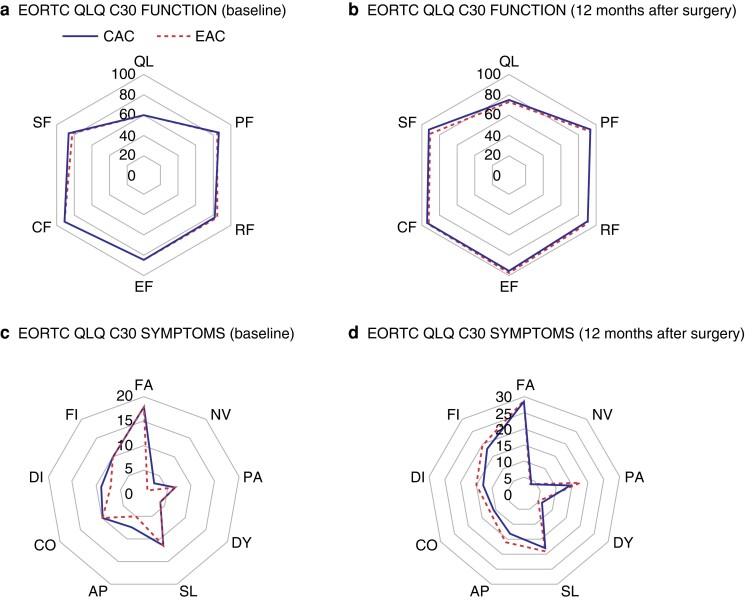
Comparison of quality of life between the early adjuvant chemotherapy arm and the conventional adjuvant chemotherapy arm **a** EORTC QLQ C30 FUNCTION (baseline). **b** EORTC QLQ C30 FUNCTION (12 months after surgery). **c** EORTC QLQ C30 SYMPTOMS (baseline). **d** EORTC QLQ C30 SYMPTOMS (12 months after surgery). EORTC QLQ, European Organization for Research and Treatment of Cancer Quality of Life Questionnaire; CAC, conventional adjuvant chemotherapy; EAC, early adjuvant chemotherapy; QL, overall health; PF, physical functioning; RF, role functioning; EF, emotional functioning; CF, cognitive functioning; SF, social functioning; FA, fatigue; NV, nausea/vomiting; PA, pain; DY, dyspnoea; SL, insomnia; AP, appetite loss; CO, constipation; DI, diarrhoea; FI, financial problem.

The QoL data are shown in *Table S2* and *Fig. 2*. There were no differences in overall health, functions, and symptoms between the two arms at any time point. These parameters were significantly improved at 12 months post-surgery compared with baseline in both arms.

## Discussion

Traditionally, AC is initiated between 4 and 8 weeks after radical surgery for colon cancer to achieve the balance between postoperative recovery and therapeutic effect. Although many studies have reported a correlation between poor prognosis and late initiation of AC (more than 8 weeks postoperatively)^4–13^, there is little research on the efficacy of immediate early initiation of AC. Moreover, the number of patients who began AC before 2 weeks was minimal (less than 0.5 per cent) in most studies. A meta-analysis by Biagi *et al*.^9^ demonstrated that both OS and DFS decreased significantly every 4 weeks, and they recommended further validation of the intuitive concept of EAC. Therefore, this study was designed to evaluate the earliest and safest timing of AC to obtain the maximal oncological effect using a prospective randomized method. As a result, it was demonstrated that EAC within 2 weeks was safe and tolerable in most patients, with equal toxicity, completeness of treatment, relative dose intensity of oxaliplatin, and QoL compared with CAC.

In the era of minimally invasive surgery and ERAS, it is necessary to re-evaluate traditional chemotherapy regimens. The traditional concept of AC after a minimum of 4 weeks after surgery was formed several decades ago when open surgery was generally performed for gastrointestinal cancers. This demanded longer hospitalization, recovery time (until returning to oral intake), and wound management. However, minimally invasive surgery for colorectal cancer has now become the standard of care for the majority of patients^18–21^ with the benefits of faster postoperative recovery and oncologically equivalent outcomes for any stage of cancer compared with open surgery^22^. Recently, several studies reported that robotic colectomy demonstrated a lower rate of conversion and a shorter hospitalization interval compared with laparoscopic colectomy^23,24^. Owing to the development of surgical techniques, the concept of ERAS has been introduced globally and has changed traditional postoperative care significantly. Changes throughout the multimodal aspects of perioperative management (including pre-admission counselling, bowel preparation, fluid therapy, nutritional support, prophylaxis against infection and thromboembolism, management during anaesthesia, and postoperative analgesia) have shortened times for return to a normal diet, recovery with less complications, and hospitalization^25^. Numerous studies have reported that ERAS significantly reduces the length of stay (to within 1 week), as well as complication and readmission rates^26–28^. Patients recover sooner from the surgical insult compared with patients historically.

Anastomotic leakage, one of the worst complications following colorectal surgery, is usually diagnosed between 3 and 8 days after surgery^22^, even though the timing of detection can vary according to a patient’s status. Additionally, anastomotic leakage after ileocolic or colocolic anastomosis is relatively uncommon compared with rectal cancer surgery and is usually detected immediately after surgery. Surgical site infection and pulmonary complications are usually reported to be diagnosed about 4 and 10 days postoperatively respectively^29^. The risk of thromboembolic events for normal patients is known to diminish after ambulation, and pharmacological prophylaxis for normal patients is recommended during admission, generally within 1 week^30^. Therefore, a minimum of 2 weeks from surgery to the initiation of AC was considered appropriate for EAC.

A potential concern after the early introduction of cytotoxic agents is late anastomotic complications. In this study, two patients in the EAC arm showed leakage on POD 16 and a stricture on POD 18 respectively, and both required surgical intervention. Although it is generally considered that anastomotic healing is completed by POD 10–12, the rate of late anastomotic leakage after general recovery was reported to be 6 to 7.7 per cent in colorectal resection, regardless of the timing of AC^31,32^. As there was no statistically significant difference in surgical complications between the two arms, it is considered that the early initiation of AC did not increase the rate of surgical complications. However, the delay of AC needs to be considered if there is evidence of surgical complications before AC.

There were five cases of recurrence during AC in the CAC arm and no cases in the EAC arm. These findings need to be evaluated further with longer-term follow-up and to investigate if patients with adverse features should be considered for earlier AC.

In the present study, postoperative QoL was investigated to evaluate if a shorter interval between surgery and following AC impairs general condition and daily living according to a patient’s subjective feelings, in addition to surgical complications. AC affects the QoL of patients who undergo colon cancer surgery, not only during AC, but also after its completion^33,34^. The impaired QoL is mainly related to chemotherapeutic toxicity and its grade during AC^35^. Iveson *et al*.^34^ reported that QoL declined during AC and started recovering 1 month after the last AC. Therefore, QoL was investigated for 1 year after surgery. In the present study, there was no statistically significant difference in QoL and its recovery pattern between the two arms. Both were worst during AC and fully recovered at 12 months after surgery. This study demonstrated that EAC within 2 weeks did not compromise surgical, medical, mental, or functional outcomes.

This study has limitations. The time interval between the two arms according to the study design was only 2 weeks. Due to the short interval between the two arms and various medical and personnel issues, there was an overlap of the time interval. Consequently, interpretation of the results should be made with some caution as 58 patients (25 in the EAC arm and 33 in the CAC arm) were excluded from the safety analyses because of protocol violations. Among them, 15 patients were enrolled in the study by surgeons immediately after recovery from surgery, but all declined to receive chemotherapy after discussions with medical oncologists. Another 40 patients chose a chemotherapy regimen other than FOLFOX. However, the outcomes of variables (chemotherapy dose, compliance, and toxicities) analysed in the safety population were similar when analyses including those patients were performed.

This study was performed in tertiary hospitals with sub-specialist colorectal surgeons, high rates of minimally invasive surgery, and low rates of surgical complications. This might impact the generalizability of the results to other centres nationally or internationally. Early initiation of AC is not appropriate for all patients and may not be appropriate for those who have open surgery, frail patients, or those with postoperative complications.

## Supplementary Material

zrad064_Supplementary_Data

## Data Availability

The data are available upon contact with the authors.
