# Supplementary material for: Short-term outcomes of Early versus conventional adjuvant chemotherapy in stage III colon cancer: randomized clinical trial
Source: BJS Open. 2023 Jul 13;7(4):zrad064. doi: 10.1093/bjsopen/zrad064 (PMC10338899; doi:10.1093/bjsopen/zrad064)
Supplement: zrad064_Supplementary_Data [file zrad064_supplementary_data.docx]

**Early vs Conventional adjuvant chemotherapy in stage III colon cancer: Short-term outcomes of a multicentre, randomized, open-label, phase III trial**

Kyung Ha Lee, Ph.D^1^, Soo Yeun Park, M.D^2^., Seung Ho Song, M.D.^2^, Hye Jin Kim, M.D.^2^, Jong Gwang Kim, Ph.D^3^, Byung Woog Kang, Ph.D^3^, In Kyu Lee, Ph.D^4^, Yoon Suk Lee, Ph.D^4^, So Hyun Kim, M.D.,^5^ Seong Kyu Baek, Ph.D,^6^ Sung Uk Bae, Ph.D,^6^ Gyung Mo Son, Ph.D^7^, Ki Beom Bae, Ph.D^8^, Gyu-Seog Choi, Ph.D^2^, Jun Seok Park, Ph.D^2^, Ji Yeon Kim, Ph.D^1^

^1^Department of Surgery, Chungnam National University Hospital, Chungnam National University College of Medicine, Daejeon, Korea, ^2^Colorectal Cancer Center, Kyungpook National University Medical Center, School of Medicine, Kyungpook National University, Daegu, Korea, ^3^Department of Oncology/Hematology, Kyungpook National University Medical Center, Kyungpook National University School of Medicine, Daegu, Korea, ^4^Department of Surgery, Seoul St. Mary’s Hospital, College of Medicine, The Catholic University of Korea, Seoul, Korea, ^5^Division of Colon and Rectum, Department of Surgery, College of Medicine, Yeungnam University, Daegu, Korea, ^6^Department of Surgery, School of Medicine, Dongsan Medical Center, Keimyung University, Daegu, Korea, ^7^Department of Surgery, Busan National University, Busan, Korea, ^8^Department of Surgery, Busan Paik Hospital, Inje University, Busan, Korea

Corresponding Author:

Jun Seok Park, M.D., Ph.D.

Colorectal Cancer Center, Kyungpook National University Medical Center, School of Medicine, Kyungpook National University, Daegu, Korea

Phone: +82-53-200-2644

FAX: +82-53-200-2027

Email: [parkjs0802@knu.ac.kr](mailto:parkjs0802@knu.ac.kr)

**Supplementary Materials – Index**

| **Supplementary Figures and Tables** |  |
| --- | --- |
| Table S1. Adverse events (Safety population)  Table S2. EORTC-QLQ C30 | *pag. 3,4*  *pag. 5,6* |

**Supplementary Figures and Tables**

Table S1. Adverse events (Safety population)

|  | EAC  (N=192) | CAC  (N=188) | P-value |
| --- | --- | --- | --- |
| All events  G0  G1-2  G3-4 | 1 (0.5)  137 (71.4)  54 (28.1) | 5 (2.7)  130 (69.1)  53 (28.2) | 0.244 |
| Anaemia  G0  G1-2  G3-4 | 141 (73.4)  51 (26.6)  0 (0.0) | 138 (73.4)  49 (26.1)  1 (0.5) | 0.597 |
| Non-febrile neutropenia  G0  G1-2  G3-4 | 11 (5.7)  150 (78.1)  31 (16.1) | 18 (9.6)  136 (72.3)  34 (18.1) | 0.291 |
| Febrile neutropenia  G0  G1-2  G3-4 | 169 (88.0)  15 (7.8)  8 (4.2) | 170 (90.4)  10 (5.3)  8 (4.3) | 0.618 |
| Thrombocytopenia  G0  G1-2  G3-4 | 76 (39.6)  113 (58.9)  3 (1.6) | 82 (43.6)  104 (55.3)  2 (1.1) | 0.684 |
| Anorexia  G0  G1-2  G3-4 | 70 (36.5)  118 (61.5)  4 (2.1) | 72 (38.3)  112 (59.6)  4 (2.1) | 0.931 |
| Nausea  G0  G1-2  G3-4 | 54 (28.1)  129 (67.2)  9 (4.7) | 58 (30.9)  123 (65.4)  7 (3.7) | 0.781 |
| Dyspepsia  G0  G1-2  G3-4 | 157 (81.8)  31 (16.1)  4 (2.1) | 154 (81.9)  31 (16.5)  3 (1.6) | 0.937 |
| Diarrhea  G0  G1-2  G3-4 | 132 (68.8)  55 (28.6)  5 (2.6) | 119 (63.3)  63 (33.5)  6 (3.2) | 0.531 |
| Vomiting  G0  G1-2  G3-4 | 144 (75.0)  46 (24.0)  2 (1.0) | 151 (80.3)  34 (18.1)  3 (1.6) | 0.346 |
| Fatigue  G0  G1-2  G3-4 | 154 (80.2)  38 (19.8)  0 (0.0) | 164 (87.2)  24 (12.8)  0 (0.0) | 0.064 |
| Constipation  G0  G1-2  G3-4 | 144 (75.0)  47 (24.5)  1 (0.5) | 138 (73.4)  46 (24.5)  4 (2.1) | 0.387 |
| Mucositis  G0  G1-2  G3-4 | 165 (85.9)  27 (52.9)  0 (0.0) | 163 (86.7)  24 (12.8)  1 (0.5) | 0.564 |
| Sensory neuropathy  G0  G1-2  G3-4 | 104 (54.2)  86 (44.8)  2 (1.0) | 117 (52.9)  70 (37.2)  1 (0.5) | 0.260 |
| Abnormal hepatic profile  G0  G1-2  G3-4 | 47 (24.5)  144 (75.0)  1 (0.5) | 57 (30.3)  130 (69.1)  1 (0.5) | 0.442 |
| Skin rash  G0  G1-2  G3-4 | 182 (94.8)  9 (4.7)  1 (0.5) | 179 (95.2)  9 (4.8)  0 (0.0) | 0.612 |
| Alopecia  G0  G1-2  G3-4 | 171 (89.1)  21 (10.9)  0 (0.0) | 178 (94.7)  10 (5.3)  0 (0.0) | 0.045 |

EAC, early chemotherapy. CAC, conventional chemotherapy

Table S2. EORTC-QLQ C30

|  | Time | Baseline | | 1 months | | 3 months | | 6 months | | 12 months | | P-value^†^ |
| --- | --- | --- | --- | --- | --- | --- | --- | --- | --- | --- | --- | --- |
|  |  | **N** | **Mean (SEM)** | **N** | **Mean (SEM)** | **N** | **Mean (SEM)** | **N** | **Mean (SEM)** | **N** | **Mean (SEM)** |  |
| **EORTC QLQ C30 FUNCTION: higher scores = better function (0-100)** | | | | | | | | | | | | |
| Global health status | EAC | 148 | 59.8 (1.3) | 138 | 58.6 (1.5) | 133 | 58.0 (1.5) | 124 | 61.2 (1.5) | 105 | 76.5 (1.5) |  |
|  | CAC | 132 | 59.1 (1.5) | 124 | 58.5 (1.2) | 126 | 58.9 (1.4) | 115 | 60.4 (1.7) | 99 | 75.3 (1.5) |  |
|  | P value* |  | 0.723 |  | 0.959 |  | 0.662 |  | 0.724 |  | 0.573 | 0.966 |
| Physical functioning | EAC | 148 | 85.1 (1.2) | 138 | 86.4 (1.1) | 133 | 86.0 (1.3) | 124 | 84.9 (1.5) | 105 | 93.6 (1.1) |  |
|  | CAC | 132 | 86.2 (1.2) | 124 | 86.1 (1.4) | 126 | 86.9 (1.2) | 115 | 87.6 (1.2) | 99 | 95.0 (0.9) |  |
|  | P value* |  | 0.518 |  | 0.865 |  | 0.612 |  | 0.165 |  | 0.329 | 0.843 |
| Role functioning | EAC | 148 | 83.2 (1.8) | 138 | 78.4 (1.9) | 133 | 77.2 (1.9) | 124 | 79.6 (2.0) | 105 | 92.2 (2.1) |  |
|  | CAC | 132 | 81.7 (1.9) | 124 | 78.5 (1.9) | 126 | 77.6 (1.9) | 115 | 80.4 (2.0) | 99 | 91.6 (2.2) |  |
|  | P value* |  | 0.310 |  | 0.970 |  | 0.882 |  | 0.778 |  | 0.844 | 0.975 |
| Emotional functioning | EAC | 148 | 84.0 (1.5) | 138 | 83.0 (1.3) | 133 | 82.4 (1.4) | 124 | 85.1 (1.5) | 105 | 94.0 (1.1) |  |
|  | CAC | 132 | 81.7 (1.7) | 124 | 80.8 (1.9) | 126 | 81.8 (1.4) | 115 | 84.1 (1.8) | 99 | 93.6 (1.4) |  |
|  | P value* |  | 0.310 |  | 0.332 |  | 0.762 |  | 0.668 |  | 0.821 | 0.956 |
| Cognitive functioning | EAC | 148 | 91.3 (1.0) | 138 | 90.3 (1.1) | 133 | 88.3 (1.3) | 124 | 87.1 (1.5) | 105 | 92.1 (1.1) |  |
|  | CAC | 132 | 91.6 (1.1) | 124 | 91.0 (1.2) | 126 | 88.8 (1.3) | 115 | 90.1 (1.2) | 99 | 93.9 (1.0) |  |
|  | P value* |  | 0.840 |  | 0.840 |  | 0.786 |  | 0.123 |  | 0.229 | 0.740 |
| Social functioning | EAC | 148 | 82.5 (1.9) | 138 | 79.5 (2.0) | 133 | 81.0 (1.9) | 124 | 81.6 (1.9) | 105 | 91.1 (1.4) |  |
|  | CAC | 132 | 84.5 (1.9) | 124 | 84.1 (1.9) | 126 | 83.9 (1.8) | 115 | 85.4 (1.7) | 99 | 93.1 (1.4) |  |
|  | P value* |  | 0.458 |  | 0.099 |  | 0.270 |  | 0.140 |  | 0.314 | 0.934 |
| **EORTC QLQ C30 SYMPTOMS: higher scores = worse symptoms (0-100)** | | | | | | | | | | | | |
| Fatigue | EAC | 148 | 28.4 (1.5) | 138 | 33.6 (1.8) | 133 | 35.3 (2.0) | 124 | 32.4 (1.9) | 105 | 16.7 (2.0) |  |
|  | CAC | 132 | 27.9 (1.8) | 124 | 30.5 (2.0) | 126 | 34.7 (2.0) | 115 | 30.7 (2.0) | 99 | 16.7 (2.1) |  |
|  | P value* |  | 0.830 |  | 0.249 |  | 0.832 |  | 0.538 |  | 1.000 | 0.924 |
| Nausea/vomiting | EAC | 148 | 3.4 (1.1) | 138 | 16.0 (1.2) | 133 | 15.8 (1.2) | 124 | 10.6 (1.2) | 105 | 1.0 (1.3) |  |
|  | CAC | 132 | 3.5 (1.2) | 124 | 15.8 (1.2) | 126 | 15.5 (1.2) | 115 | 10.4 (1.3) | 99 | 3.0 (1.4) |  |
|  | P value* |  | 0.950 |  | 0.907 |  | 0.859 |  | 0.910 |  | 0.296 | 0.880 |
| Pain | EAC | 148 | 17.7 (1.3) | 138 | 11.7 (1.4) | 133 | 11.3 (1.4) | 124 | 10.8 (1.6) | 105 | 5.7 (1.2) |  |
|  | CAC | 132 | 14.2 (1.5) | 124 | 10.6 (1.5) | 126 | 12.0 (1.8) | 115 | 10.3 (1.4) | 99 | 5.1 (1.3) |  |
|  | P value* |  | 0.078 |  | 0.592 |  | 0.757 |  | 0.815 |  | 0.735 | 0.641 |
| Dyspnea | EAC | 148 | 5.0 (1.1) | 138 | 5.6 (1.1) | 133 | 9.3 (1.7) | 124 | 10.8 (2.0) | 105 | 3.5 (1.1) |  |
|  | CAC | 132 | 6.5 (1.4) | 124 | 6.2 (1.3) | 126 | 7.7 (1.5) | 115 | 8.4 (1.6) | 99 | 3.0 (1.0) |  |
|  | P value* |  | 0.396 |  | 0.723 |  | 0.483 |  | 0.354 |  | 0.738 | 0.646 |
| Insomnia | EAC | 148 | 18.9 (2.1) | 138 | 22.2 (2.2) | 133 | 22.8 (2.2) | 124 | 23.7 (2.3) | 105 | 10.8 (1.7) |  |
|  | CAC | 132 | 18.0 (2.3) | 124 | 22.3 (2.4) | 126 | 22.5 (2.2) | 115 | 20.9 (2.2) | 99 | 10.4 (1.8) |  |
|  | P value* |  | 0.772 |  |  |  | 0.923 |  | 0.381 |  | 0.872 | 0.971 |
| Appetite loss | EAC | 148 | 16.2 (1.8) | 138 | 28.7 (2.2) | 133 | 29.3 (2.3) | 124 | 25.0 (2.2) | 105 | 4.1 (1.3) |  |
|  | CAC | 132 | 13.0 (1.8) | 124 | 24.2 (2.2) | 126 | 29.9 (2.2) | 115 | 23.8 (2.5) | 99 | 7.1 (26.8) |  |
|  | P value* |  | 0.221 |  | 0.150 |  | 0.851 |  | 0.718 |  | 0.872 | 0.422 |
| Constipation | EAC | 148 | 12.1 (1.7) | 138 | 15.2 (1.8) | 133 | 13.8 (1.9) | 124 | 14.0 (2.1) | 105 | 8.9 (2.0) |  |
|  | CAC | 132 | 10.5 (1.9) | 124 | 9.9 (1.7) | 126 | 14.0 (1.9) | 115 | 9.0 (1.7) | 99 | 9.1 (1.8) |  |
|  | P value* |  | 0.530 |  | 0.034 |  | 0.941 |  | 0.068 |  | 0.941 | 0.376 |
| Diarrhea | EAC | 148 | 14.8 (1.7) | 138 | 14.7 (1.9) | 133 | 13.0 (1.7) | 124 | 11.6 (1.8) | 105 | 6.7 (1.5) |  |
|  | CAC | 132 | 13.0 (2.0) | 124 | 14.5 (2.0) | 126 | 11.6 (1.7) | 115 | 14.8 (1.9) | 99 | 8.4 (1.5) |  |
|  | P value* |  | 0.491 |  | 0.942 |  | 0.561 |  | 0.222 |  | 0.424 | 0.602 |
| Financial problem | EAC | 148 | 19.4 (2.2) | 138 | 18.1 (2.0) | 133 | 15.3 (2.1) | 124 | 17.2 (2.2) | 105 | 7.9 (1.7) |  |
|  | CAC | 132 | 17.5 (2.2) | 124 | 16.4 (2.2) | 126 | 13.2 (1.7) | 115 | 13.0 (1.9) | 99 | 7.7 (1.7) |  |
|  | P value* |  | 0.543 |  | 0.567 |  | 0.441 |  | 0.153 |  | 0.934 | 0.929 |

SEM, standard error of mean

^*^Comparison between both arms at each time periods

^†^Comparison between group by time interaction
